# Performance after training in a complex cognitive task is enhanced by high-definition transcranial random noise stimulation

**DOI:** 10.1038/s41598-022-08545-x

**Published:** 2022-03-17

**Authors:** Quentin Chenot, Caroline Hamery, Evelyne Lepron, Pierre Besson, Xavier De Boissezon, Stéphane Perrey, Sébastien Scannella

**Affiliations:** 1 ISAE-SUPAERO, Université de Toulouse, Toulouse, France; 2 EuroMov Digital Health in Motion, Univ Montpellier, IMT Mines Ales, Montpellier, France; 3 Toulouse Neuroimaging Center (ToNIC), Université de Toulouse, INSERM, Toulouse, France; 4 Department of Physical Medicine and Rehabilitation, University Hospital of Toulouse, Toulouse, France

**Keywords:** Neuroscience, Psychology

## Abstract

Interest for neuromodulation, and transcranial random noise stimulation (tRNS) in particular, is growing. It concerns patients rehabilitation, but also healthy people who want or need to improve their cognitive and learning abilities. However, there is no consensus yet regarding the efficacy of tRNS on learning and performing a complex task. In particular, the most effective electrode montage is yet to be determined. Here, we examined the effect of two different tRNS montages on learning rate, short- and long-term performance in a video game (Space Fortress) that engages multiple cognitive abilities. Sixty-one participants were randomly assigned to one of three groups (sham vs. simple-definition tRNS vs. high-definition tRNS) in a double-blind protocol. Their performance on the Space Fortress task was monitored during a 15-day experiment with baseline (day 1), stimulation (day 2 to 4), short- (day 5) and long-term (day 15) evaluations. Our results show that the high-definition tRNS group improved more on the long term than simple-definition tRNS group, tended to learn faster and had better performance retention compared to both simple-definition tRNS and sham groups. This study is the first to report that high-definition tRNS is more effective than conventional simple-definition tRNS to enhance performance in a complex task.

## Introduction

During the last 10 years, the number of publications studying the effects of the transcranial electrical stimulation (tES) on cognitive training and cognitive functions has blossomed^1^. Here, we sought to investigate the effect of tES on the learning and retention of cognitive skills in a video-game like complex task. More specifically, we assessed two different montages (large pad-electrodes in a bilateral configuration vs. multi smaller electrodes in a $4 \times 1$ ring configuration) of a particular tES technique—the transcranial random noise stimulation (tRNS).

The effects of tES, especially transcranial direct current stimulation (tDCS) on cognitive performance has been widely studied^2^. Nevertheless, the results are mostly heterogeneous with some studies showing an increase of performance whereas other studies show no improvement^2–4^. The reasons for these heterogeneous results may be found in the limits of the tDCS, which delivers a direct electrical current with a constant intensity and fixed polarity^1^. This constant intensity may be a limit as it could prevent persistent changes in neuronal membrane potentials through homeostatic mechanisms^5,6^.

Recently, a focus has been emphasized on tRNS that delivers a low intensity current at random frequencies, producing noise in the neural system. While the precise effect of such noise is still debated^1^, the proposed mechanisms include enhanced cortical excitability^7^, lower inhibitory thresholds in neurons^8^, facilitation of sodium channels reopening (i.e. repolarization) after a depolarization^9^ and stochastic resonance^10^. Stochastic resonance refers to the amplification of a weak signal with the addition of noise^11^. More specifically, it has been suggested that this phenomenon modulates, not only the activity of the neurons under the site of stimulation, but also the activity of the other distant, inter-connected, neurons^12^. By inducing noise, such stimulation may modify the discharge threshold of the neurons in the related brain networks, which should in turn induce a modification of behavioral performance^1^.

These potential behavioral modifications have been investigated, and a summary of methods and results of studies (as of July 2021) that used tRNS and measured its effects on cognitive functions can be found in Table S2 in supplementary material. These include a wide range of high-level cognitive functions, spreading from attention^13^, working memory^6,14^, inhibition^15–17^, to mathematical skills^18–21^ and multitasking^22^. Noteworthy, the effects of tRNS on high-level cognitive functions are heterogeneous across studies with some studies showing no effects^15,23,24^ and several others showing significant effects^6,18,21,22,25–27^. For instance, Brem et al.^25^ showed that groups that received either tRNS or tDCS coupled with cognitive training (by means of a video game) had better transfer effect on fluid intelligence tasks compared to a sham stimulation. In the same way, another study^26^, using an executive function training protocol coupled with tES, showed that the tRNS group had better improvements than the sham group. In addition to longitudinal, multi-session studies, Harty et al.^22^ investigated the effect of tRNS on learning of a complex and ecological task (start-up procedure of simulated waste water treatment) during one learning session. They showed that tRNS promoted better multitasking performance and better long-term retention of the task during the second session (after 2 weeks) compared to the sham group. Taken together, these results point out that tRNS is promising for training complex task skills that involve multiple cognitive functions.

Several studies presented above used a conventional bilateral tRNS montage over the dorsolateral prefrontal cortex (DLPFC), with large pad electrodes (typically between 25–35 cm^2^), soaked with saline and placed on F3 and F4 scalp positions of the 10–20 international system^20–23,26^. We will refer to this montage as SD (simple definition). Recent development in tES have also used a $4 \times 1$ montage (i.e. 4 peripheral and 1 central electrode), to which we will refer as HD (high definition). This type of montage has the advantage of being more focal thanks to the use of smaller electrodes (e.g. $\pi$ cm^2^) distributed over a predetermined brain area. This HD-montage has been used with various types of stimulation, including tDCS^28^, but also transcranial alternating current stimulation^29–32^ (tACS). Such HD-montage is now thought to be one the leads to optimize the stimulation effects on a specific area, regardless the type of stimulation^28,33^.

Specifically, both right and left DLPFC are typically thought to be recruited when performing complex tasks. It has been shown higher activity in these areas when individuals perform tasks involving executive functions tasks^34–38^ or multitasking^39,40^. Especially, the right DLPFC seems to be more involved during planing^41^, a key component of executive functions^42^. Therefore, a focal stimulation with the latter area may be more efficient than a conventional SD montage to improve training and cognitive performance in complex tasks.

**Figure 1:**
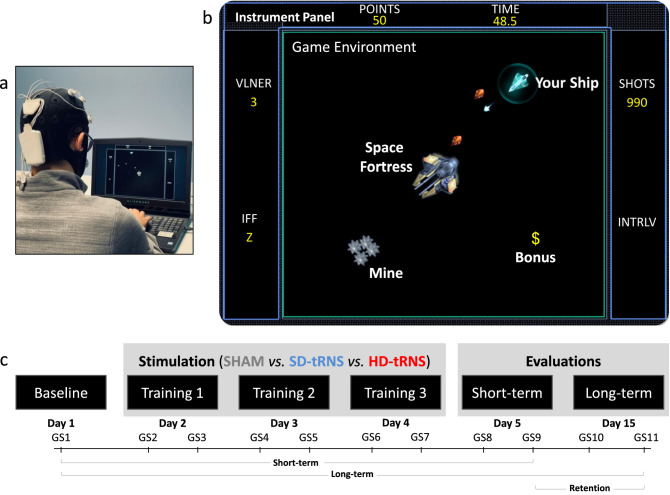
Experimental design and performance evaluation. (**a**) A participant playing the Space Fortress game (in this example, the participant is equipped with a $4\times 1$ HD montage). (**b**) Space Fortress in-game screenshot. (**c**) Timeline of the experimental protocol. GS*n* stands for Game Session number *n* across days. Short-term is the delta of performance between GS9 and GS1. Long-term is the delta of performance between GS11 and GS1. Retention is the delta of performance between GS11 and GS9.

However, to our knowledge, no study has attempted to investigate the potential advantages of HD-tRNS over a standard SD-tRNS montage during a 1-week training protocol of a complex task. In the present study, we used the Space Fortress (SF) video game as a complex task (Fig. 1). We selected this game because it has been specifically designed by psychologists to study complex task learning^43^. In this 2D game, the player’s performance is assessed through the practice of four concomitant sub-tasks: controlling a ship in the space, destroying a fortress, destroying moving mines and capturing bonuses (see Table S1 in supplementary material). Because of the multitasking skills required to perform well in this game, it makes it an interesting task to learn and master, necessarily involving several high-level cognitive processes such as executive functions^44,45^. This assertion is supported by the fact that SF training has been shown to modify the activity and the connectivity of the fronto-parietal network^46^, which is typically involved in high-level cognitive functions^47–49^. Therefore, we aimed to stimulate a key brain region in this network—the DLPFC^50^—in order to drive brain plasticity during SF learning.

Based on previous literature that showed that tRNS may increase performance in complex cognitive tasks^21,22,25,26^, we expected (hypothesis 1) that the stimulated groups (SD-tRNS and HD-tRNS) would have a better learning rate in the SF task compared to the control group (sham). This higher learning rate would result in higher performance during both short-term, that is the day right after the training, (hypothesis 2.a.) and long-term evaluation, 10 days after the training (hypothesis 2.b.). We expected that the stimulated groups would also have better retention of performance between the end of the training and ten days after (hypothesis 3). Finally, based on the studies that showed that HD-tDCS may have an increased effect compared to SD-tDCS^51–54^, we expected the HD-tRNS group to exhibit higher effects than the SD-tRNS group for all variables mentioned in the previous hypotheses (hypothesis 4).

To test these hypotheses, we ran a double-blind, randomized, placebo-controlled experiment. The experimentation lasted for five consecutive days at a fixed schedule, with one additional session ten days later. Day 1 was the baseline for SF performance, with no stimulation. From days 2 to 4 (training period), participants played two game sessions a day, each lasting 10 min. The stimulation was performed according to the group assignment (sham vs. SD-tRNS vs. HD-tRNS). The last two experimental sessions (short and long-term evaluations) were similar, but with no stimulation (see Fig. 1). To assess the effect of the stimulation, we computed the learning rate as the regression coefficient of a logarithmic adjustment model of SF performance over time^55^. We also measured the deltas of performance between baseline and short-term, baseline and long-term, and between short-term and long-term (retention) experimental sessions. One way ANCOVAs (analyses of covariance) were conducted to investigate differences between groups (sham vs. SD-tRNS vs. HD-tRNS) on the learning rate and on the deltas of performance, each time controlling for video game experience (the covariate). Gaming experience was established with a standardised questionnaire to assess the frequency and intensity participants used to and/or presently play video games.

## Results

### Assessment of individual learning rates

**Figure 2:**
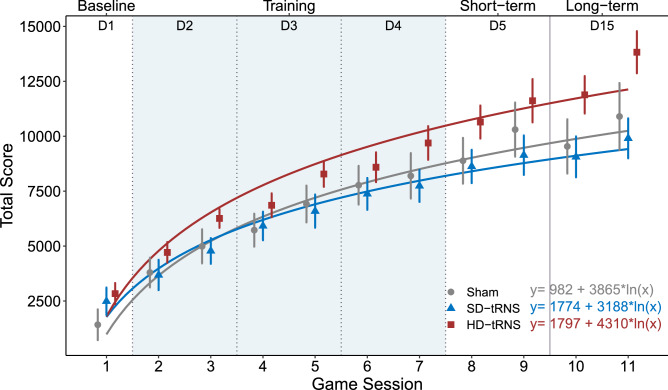
Mean total scores for Space Fortress game (with standard error bars) per group across game sessions. The fitted ln model across game sessions and for each group is presented in the lower right part. *D* day.

**Figure 3:**
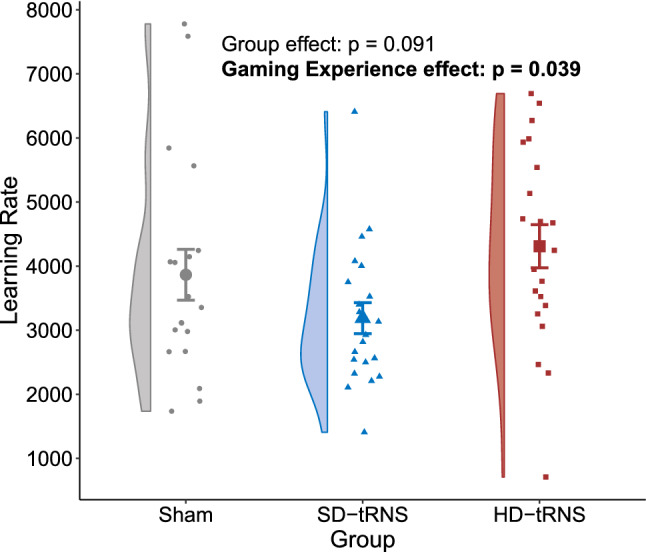
Learning rate by stimulation group (Hypothesis 1). Bold: $p < 0.05$.

The ANCOVA on the learning rates (Figs. 2 and 3) showed a significant effect of the gaming experience ($F_{(1,57)}=4.47$, $p=0.039$, $\eta _p^2=7\%$, $b=0.26$, power $=0.55$), whereas the group factor did not reach significance ($F_{(2,57)}=2.50$, $p=0.091$, $\eta _p^2=8\%$, power $=0.48$)

### Behavioural effects of the stimulation type

Except for day 1, when the baseline was recorded, participants performed two game sessions (GS) each day, leading to a total of 11 GS. We computed the performance improvement as the difference of scores between the baseline score at day 1 (GS1) and the second game session of day 5 for the short-term delta score (GS9–GS1), and between GS1 and the second game session of day 15 for the long-term delta score (GS11–GS1). The retention delta score was computed as the difference between the long-term (day 15) and short-term (day 5) second game sessions (GS11–GS9). Figure 1 gives an overview of days and game sessions distribution over time, and illustrates the timeline for the delta scores.

**Figure 4:**
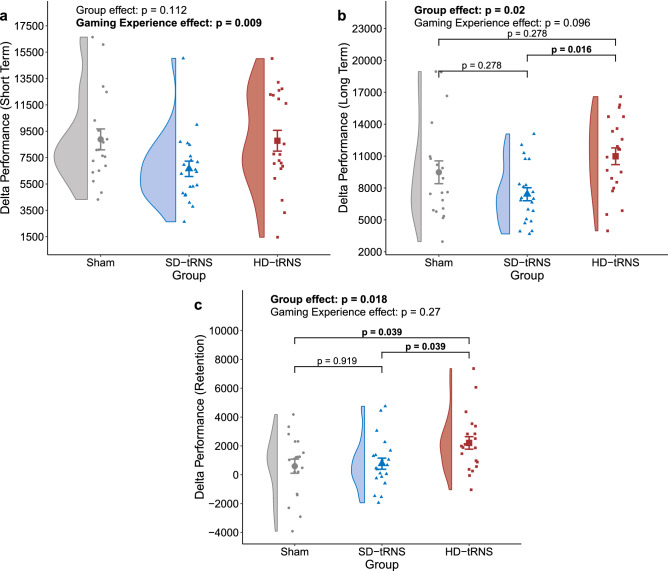
(**a**) Delta between short-term and baseline performance (Hypothesis 2.a.). (**b**) Delta between long-term and baseline performance (Hypothesis 2.b.). (**c**) Delta between long-term and short-term performance (Hypothesis 3.). *GS* Game Session. Bold: $p < 0.05$.

The short-term delta score was significantly and positively influenced by the gaming experience ($F_{(1,57)}=7.37$, $p= 0.009$, $\eta _p^2=11\%$, $b=0.36$, power $=0.76$) but not by the stimulation group ($F_{(2,57)}=2.27$, $p= 0.112$, $\eta _p^2=7\%$, power $=0.44$) (Fig. 4a).

The gaming experience effect faded with time to become non significant for both the long-term delta score ($F_{(1,57)}=2.87$, $p= 0.096$, $\eta _p^2=5\%$, $b=0.20$, power $=0.38$) and the retention delta score ($F_{(1,57)}=1.24$, $p= 0.270$, $\eta _p^2=2\%$, $b=-0.14$, power $=0.19$). However, significant effects of the stimulation group were found for both long-term delta ($F_{(2,57)}=4.21$, $p= 0.020$, $\eta _p^2=13\%$, power $=0.72$) and retention delta ($F_{(2,57)}=4.32$, $p= 0.018$, $\eta _p^2=13\%$, power $=0.73$) (Fig. 4b,c).

### tRNS Adverse Effects Questionnaire

The results of the tRNS Adverse Effects Questionnaire can be found in Figs. S2 and S3 in supplementary material. A Kruskal–Wallis test compared the three groups on the post session questionnaire and revealed a significant effect ($p=0.034$). Post-hoc analyses showed that the HD-tRNS group had higher scores than the SHAM group ($p=0.048$).

## Discussion

The present experiment is the first to investigate the effects of different tRNS montages (SD vs. $4\times 1$ HD) on learning a complex video game during a one-week training. We hypothesized that both tRNS groups would have a better video game performance compared to the sham group, with an additional advantage for HD stimulation. More specifically, we excepted higher learning rates and improvements on the short-term (day 5 vs. day 1), the long-term (day 15 vs. day 1) and on the retention period (day 15 vs. day 5). Although no short-term effect was observed, our data partially support our hypotheses with a tendency for the learning rate, along with a significant long-term effect for HD compared to SD, but not to sham. In addition, the highest retention performance was observed for the HD-tRNS group, compared to both sham and SD groups this time. In contrast, the SD-tRNS montage showed no differences with the sham group, whatever the comparison. To our knowledge, our study is the first to provide evidence that an HD-tRNS montage may be more effective than an SD-tRNS montage to train complex task skills in healthy adults. This study also confirms that the right DLPFC is a valid target for neuromodulation and enhancement of high-level cognitive functions^23^.

One important finding here is the positive effect of the neurostimulation, for the HD-montage only, during the retention period—10 days after the end of training and stimulation. The difference between the stimulation groups was indeed the strongest between day 5 and day 15, suggesting that HD-tRNS would have an outlasting effect on performance gain once the training is over^56^. This effect was not abrupt, but rather it seems to have strengthened over time. This observation is supported by the absence of short-term effect, followed by a superiority of HD montage that became significant for the long-term (compared to SD only), and for retention period (compared to both sham and SD).

This delayed behavioral effect might be explained by the time necessary to modify the activity of the fronto-parietal network, solicited while playing SF video game^57^. Indeed, one of the main action principle of tRNS is supposed to occur through stochastic resonance^1^. According to this hypothesis, by inducing noise, such stimulation would modify the discharge threshold of the neurons in the targeted brain networks. Simultaneous neuronal stimulation would induce subsequent synaptic strengthening according to the coincidence-detection rule proposed by Hebb^58^ and Konorski^59^. Long-term potentiation (LTP) has been proposed as one of the main consequences of this synaptic modification, to explain how the memory and the learning are stored in the brain^60,61^ following cognitive training and brain stimulation. More specifically, it has been proposed that cellular changes might be responsible for the long-term effects of tRNS^62^. On a more marcroscopic view, it has been shown that tRNS combined with cognitive training induced more efficient neurovascular coupling^21^. These neural mechanisms take place as the cognitive training starts, but may be measurable with some delay. The low intensity (from − .5 to .5 mA) of the tRNS and the few stimulation sessions (a total of 60 min) in our study, arguably limited the extent of such synaptic plasticity, both in time and intensity, which may have led to a delayed observation of these effects. It seems therefore important to extend the classical pre-post evaluation of cognitive training with additional long-term and retention assessments in future studies.

**Figure 5:**
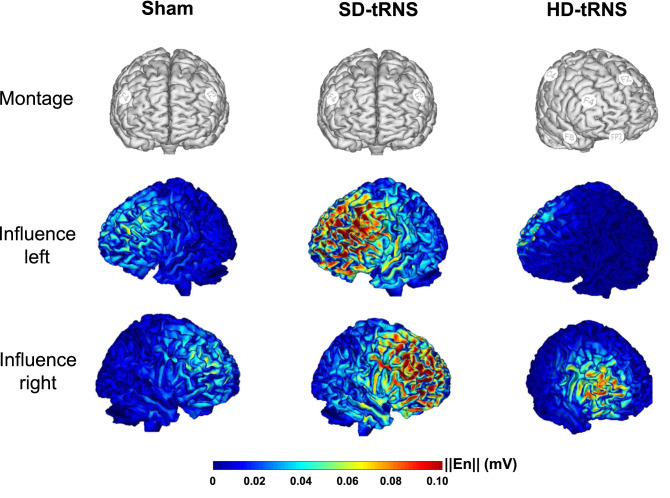
Stimulation montages and influence maps according to the montage type. Extracted from NIC2 software v.2.0.11.7.

Another important result for future research in non-invasive neurostimulation is the superiority of the $4\times 1$ HD-tRNS montage over the SD montage. Although the actual mechanisms of tRNS are still discussed^1^, this difference might lie in the distribution of the current flow, as illustrated in Fig. 5. First, the stimulation is more focal in the HD montage; the target area is narrowed and, therefore, receives a greater amount of current that may induce higher stochastic resonance effect^1^. Second, the HD stimulation only targeted the right hemisphere, while the SD targeted both left and right. The right and left DLPFCs have been described as crucial for multitasking^39,40^ and executive functions^37,38^ in general, but it has also been suggested that concomitant stimulation of two interconnected brain areas might be more disruptive than beneficial because of inter-hemispheric inhibition^63,64^. The rapid (i.e. up to 2 ms) alternating stimulation of left and right DLPFCs with the SD montage may have induced such inter-hemispheric inhibition in the present study, which could have lead to an absence of behavioral difference with the Sham group. This superiority of unilateral over bilateral montage has been previously observed in a tDCS methodological article^65^. The authors measured a higher electrical dose within the targeted DLPFC and also within the connected network nodes (i.e. the ventral attention network, the limbic system and the executive control network).

Although the superiority of the HD montage was among our hypotheses, the non-significant difference between the SD-tRNS and the sham groups was not expected. From the few other studies that have used similar tRNS parameters (i.e. F3/F4 electrode position, frequency range, intensity range, stimulation duration), three did show significant effects of the stimulation^20,21,26^ and one did not^23^. Several factors such as lack of robustness in experimental designs, absence of full details about stimulation parameters, suitability of statistical approaches, and publication in favor of positive results^66^ have been proposed to explain the discrepancy of results^23^. Specifically, the present study (stimulation parameters) was designed based on Snowball et al.^21^ who found an improved learning rate and long-term performance in an arithmetic task for the tRNS group compared to a sham group. However, our study differs in the following points: lower frequency range (100–500 vs. 100–600 Hz), shorter training protocol (3 consecutive days of coupling tRNS and cognitive training vs. 5 consecutive days) and a different experimental task (Space Fortress vs. arithmetic task). All or some of these factors may explain the absence of effect found in our SD-tRNS group. Meanwhile, taking into account the superiority of the HD montage, we would recommend to rather consider this montage option over the SD one for a similar investigation. Further studies will be necessary to determine the tRNS parameters, brain location and/or cognitive training types most sensitive to the SD montage.

As expected, video game experience had a significant role during SF training^45,67^. Participants who were more exposed to video games (e.g. number of played hours per week) were more likely to perform better on SF. However, one interesting result is that the more the participants trained to the SF task, the lower this effect; eventually it became non-significant in the last session. On the contrary, the stimulation effect became more important as training went by. As a result, the gaming experience was an important variable to control for, even though its impact diminishes with training.

On average, the responses of the stimulated groups on the tRNS adverse effect questionnaire were slightly higher than those from the sham group (1.6 points higher for the HD-tRNS group, and 0.90 points higher for the SD-tRNS group compared to the 1.32 points Sham average score on the 48 point-scale, see Fig. S2 in supplementary material). This result indicates that some participants within these groups experienced an effect of the stimulation which was not present in the sham group. Looking specifically at the responses on each question (see Fig. S3 in supplementary material), we can identify that participants from the stimulated groups reported slightly higher psychological (fatigue, difficulties in concentrating) and physical (tingling, itching, flickering) symptoms. However, one should note that most participants in all groups reported no symptoms at all, especially in some important ones (pain, nausea, headache). These results are in agreement with a systematic review^68^, which showed that active stimulated groups often report slightly higher (but often non-significant) adverse effects than sham groups.

A limit lies in the moderate statistical power of the present study (ranging from 0.19 to 0.76). While being slightly higher than most of recent tRNS studies that include 10 to 18 participants per group^6,14,16,17,20,21,23–25,27,69^, this is still not enough to obtain a 0.80 power with a *p* value criteria of 0.05, as recommended^70^. This statistical power should be further improved to increase the confidence we have in the significant and non-significant results. In general, future studies should embrace the open science opportunity in order to predetermine sample size and to produce registered reports when appropriate, in order to eliminate some common statistical biases^71^. Future studies should also consider investigating the neural mechanisms of tRNS. Indeed, experimental works with animals are crucially lacking, and to date, only hypotheses about these mechanisms have been proposed, with few empirical data to support them^1,11^.

In conclusion, this study is the first to report that a $4\times 1$ high-definition tRNS montage coupled with cognitive training on a complex video game task may be efficient to promote outlasting performance gain. This result is especially important because the learning period was relatively short—with only three 20-min learning sessions coupled with tRNS. This finding may have implications for students learning a profession with either complex tasks to be performed or prolonged training, such as pilots, air traffic controllers, surgeons, or software developers. Future studies may also explore the possibility of such montage to treat patients with cognitive disorders.

## Methods

### Participants

Based on an a priori power analysis (available in supplementary material), 66 participants were initially planned. In total, 70 participants were recruited in the ISAE-SUPAERO (Toulouse, France) and Montpellier University campuses through flyers and mailing lists. Participants had to meet the following eligibility criteria and safety guidelines for tES use: to be free of a history of neurological, psychiatric or sensory disorders, cardiac or cardiovascular pathology, and no recent use of medications or substances that might interfere with brain activity.

From these 70 participants, 61 constituted the final sample. Four participants withdrew from the study (two because they were not available for the long-term session, and two because they were not psychologically comfortable with brain stimulation after the first session), and five were excluded because of impedance unstability detected by the stimulation software. All participants were given detailed information about the experiment and gave informed consent prior to start. The demographics of the sample are described in Table 1. The study was approved by the local ethic committee of EuroMov-Montpellier (IRB1805A) and performed following the Declaration of Helsinki.

**Table 1:** Participants’ demographics (mean ± SD).

	Sham	SD-tRNS	HD-tRNS	*p*-value***
N	19	21	21	NA
Gender	4 women	3 women	3 women	NA
Handedness	1 LH	1 LH	1 LH	NA
Age	$24.0 \pm 4.6$	$22.7 \pm 3.1$	$24.1 \pm 3.4$	0.39
Education$^{\text {a}}$	$16.2 \pm 2.0$	$16.4 \pm 1.6$	$16.3 \pm 1.8$	0.93
VGexp$^{\text {b}}$	$4.8 \pm 2.4$	$3.8 \pm 1.5$	$5.0 \pm 3.0$	0.32

$^{\text {a}}$Education is measured on a scale from 1 to 20 (with 12 = high school degree). $^{\text {b}}$Video Game experience (VGexp) is measured by a questionnaire and range on a scale from 0 to 10. ***ANOVA’s were performed for Age and Education, and a Kruskal-Wallis was performed for VGexp (available in Supplementary Information). *LH* Left-handed

### Design and procedure

A double-blind, randomized, placebo-controlled design was used for this study. Each participant was randomly assigned (by computer generation) to one of the three stimulation groups (Sham vs. SD-tRNS vs. HD-tRNS) using a priori randomization schedule. To ensure the double-blind, and as sham was an SD-like montage, the experimenter was led to believe that the design also included an HD-Sham condition (fictive fourth group). The experimenter was not aware of our hypothesis of a superior effect of the HD over the SD montage. The principal investigator did not take part in the experiments. He created a list wich randomly assigned each of the 66 participants to one of the three groups (HD-tRNS, SD-tRNS, Sham). To ensure the double-blind for the HD-tRNS group, the 22 participants in this group were then divided into two fictive groups in the list, HD-tRNS and HD-sham (also generated randomly). For each new participant entering the study, the experimenter was given the corresponding group, and only at this moment. In other words, he did not have access to the entire list prior to the study and was led to believe to the existence of 4 groups of 22 participants each. The double-blind option resulting in 4 fictive groups (group 1, group 2, group 3 and group 4) for the experimenter was activated at the beginning of the study in the NIC software (Neuroelectrics, Barcelona, Spain). In addition, the principal investigator was the only one to know the password to unblind the protocol. The experiment was then stopped when we reached our goal of 66 participants. At this time, we unblinded the protocol, and we checked that the experimenter was indeed not able to identify the groups and was convinced by the fictive 4-group protocol.

For each participant, the experimentation lasted for five consecutive days at a fixed schedule with one additional session ten days later. At the beginning of the first day, participants completed questionnaires to gather demographics data (age, education level, handedness and game-level). Then, they read the rules of the SF task and practiced the video game for approximately 10 min in order to get used to the game rules (see below for the detailed practice description). Finally, they performed the first game session (baseline, day 1), playing SF for 10 min. From day 2 to day 4 (training period), participants played two game sessions, each lasting 10 min. During these experimental sessions, stimulation was performed according to the group assignment (sham vs. SD-tRNS vs. HD-tRNS). The last two experimental sessions (short and long-term evaluation) were similar, but with no stimulation (see Fig. 1).

All experimental sessions were performed in an experimental room with no window and with a stable temperature. The SF task was displayed on a 17$^{\prime \prime }$ laptop. The stimulation device was systematically installed, and participants had to answer a questionnaire about potential sensations that could be related to electric stimulation (see below for details).

### Transcranial random noise stimulation

The tRNS was applied only during the training sessions (days 2, 3 and 4, see Fig. 1b). The other days, such as baseline (day 1), short-term (day 5) or long-term (day 15), the participants did not receive stimulation.

During all sessions, the participants were mounted with a neoprene head cap (see Fig. 5). The SD-tRNS and sham groups had the same montage, with two circular sponstim ($25$ cm^2^) positioned on F3 and F4 (left and right DLPFCs) according to the 10–20 system of electrode placements. The HD-tRNS group had a $4 \times 1$ montage, with 5 circular ($\pi$ cm^2^) NG Pistim gel electrodes placed on the C4, Fp2, Fz, F8 locations for the peripheral electrodes and F4, over the right DLPFC, as the central electrode.

The tRNS waves for the stimulated groups (SD-tRNS and HD-tRNS) were generated using a Starstim device (Neuroelectrics®). The random level of current was generated for every sample (sampling rate 1280 samples/s). The current intensity was randomized, between $-\,.5$ and .5 mA (mean = 0; SD = 0.333) following a normal distribution, which was in line with previous studies^21,25^. As the current distribution in large band of high frequencies has been recommended for tRNS^72^, we chose the largest possible range of current frequency allowed by our device, which was distributed between 100 and 500 Hz. In the case of SD-tRNS montage, the two electrodes always send or received 100% of the delivered current. In the HD-tRNS montage ($4 \times 1$), the “1” central electrode (F4) always send or received 100% of the delivered current, whereas the “4” peripheral electrodes send or receive about 25% (23% for F8 and Fz and 27% for C4 and Fp2) of the current, individually. Note that the percentages values have been calculated depending on the distance from F4. Both stimulated groups received a gradual rise (current ramp up) of 15 s at the beginning of stimulation and a gradual decrease (current ramp down) during the last 15 s. Between ramps, 20 min of stimulation matched the 20 min of Space Fortress training. Both stimulated groups received a total of 394.6 mC during the whole stimulation. Resulting influence maps are presented in Fig. 5. Note, however, that this total of electric charge was differently distributed in these groups due to the montages. In the HD-tRNS montage, this amount of electric charge was distributed mainly on one brain area (the right DLPFC), while in the SD-tRNS montage, it was equally distributed across both hemispheres (mainly on right and left DLPFCs). The sham group received the same current ramp up and down than the stimulated groups at the beginning and end, respectively, but no stimulation during the 20 min of SF playing. Consequently, the sham group received a total of 7 mC counting both ramp up and ramp down.

### The space fortress game

For the purpose of this study, a Python-based (ver. 2.7) version of SF was chosen from the github.com/CogWorks website (see Fig. 1). A clear overview of the game is described by Boot et al.^45^. The authors’ description highlights the complexity of this complex task and the variety of cognitive resources needed to play the game. In brief, the main goal of the game is to control a spaceship in space with no gravity (first task). The second goal is to destroy the fortress (second task). In addition, the player has to memorize three specific letters that will help him to identify and destroy different types of mines (type-1 and type-2) that regularly appear in the game (third task). Simultaneously, she/he must keep focusing on sequences of symbols (e.g. # and $) that appear continuously throughout the game in order to capture bonuses (fourth task). To obtain the higher possible score, the participant has to perform the four sub-tasks in parallel. To this goal, participants were given the following instruction: “You will maximize your score by performing all sub-tasks to the best of your abilities”. A more precise description of rules and points distribution are available in Table S1.

During the first day, participants practiced the game. This practice started with reading a document that described the task (environment and rules). After reading, participants were asked to play the SF game for approximately 10 min. They practiced one task at a time following the variable-training methods used in previous studies^45^. More precisely, participants were asked: to control the ship and only focus on destroying the fortress (step 1); to capture bonuses only (step 2); to destroy mines only (step 3); and finally to perform all tasks together (step 4). Note that participants were informed about the points distribution prior to play. The experimenter made sure that participants understood and had experienced all the rules of the SF game before starting the baseline evaluation.

A total of 11 SF game sessions (GS) of 10 min each were performed by the participants during six experimental sessions (day 1 to day 5 plus day 15, see Fig. 1). Two game sessions per experimental session were completed except for day 1 that consisted of one game session only (baseline evaluation). Participants saw their score displayed at the end of each session. One SF total score was calculated per game session and corresponded to the sum of the points that included all sub-tasks.

### Video game experience questionnaire

Participants were asked three questions to assess their video game habits: (1) “In the last 12 months, how many times have you launched a video game (PC, home or portable consoles, smartphone)” (five-point Likert scale), (2) “Currently, on average, how much time per day do you spend playing video games?” (four-point Likert scale) and (3) “Have you had a period, or periods, in your life when you played video games intensively (more than 2 h a day on average for at least 3 months)?” (yes/no). The total score of this questionnaire was computed as the sum of all responses and ranged from 0 to 10.

### tRNS Adverse Effects Questionnaire

After each session, a translated version (french) of the tDCS Adverse Effects Questionnaire^68^ was administered to the participants (renamed tRNS Adverse Effects Questionnaire in this article). This questionnaire included 12 items on how they currently evaluate different sensations (headache, difficulty to concentrate, change of mood, change in visual perception, tingling, itching, burning, pain, fatigue, nervousness, unpleasant sensations and nausea, see Fig. S3) in which the participants rated on a five-point Likert scale (from 0 to 4). The total score of this questionnaire was computed as the sum of all responses and ranged from 0 to 48.

### Statistical analyses

Statistical analyses were performed using the R studio software (R v.4.0.5^73,74^).The assumptions of normality and homoscedasticity were checked with visual inspection of residuals (normal probability plot and residuals vs. predicted values respectively). Statistical tests (Shapiro–Wilk and Levene tests respectively) were additionally run when necessary.

We computed the learning rate using a log adjustment model ($y = a + b \times ln(x)$), where *y* is SF performance, *x* is the game sessions over time, *a* is the intercept and *b* the regression coefficient)^55^. This learning rate was computed for each participant and comprised all experimental sessions (two games per session) including baseline training and short and long-term evaluations. The regression coefficient from the model is the individual learning rate.

A one-way ANCOVA was conducted to assess significant difference between groups (sham vs. SD-tRNS vs. HD-tRNS) on the learning rate (*b*) controlling for video game experience (hypothesis 1). As gaming experience is likely to influence SF performance^45,67^, we decided to add it as a co-variate to ensure that the results showed the effect of the stimulation, independently of gaming experience.

We then measured the delta of performance between the baseline and the short-term session (GS9–GS1), between the baseline and the long-term session (GS11–GS1) and between the short-term and the long-term sessions (GS11–GS9) corresponding to the retention period. ANCOVAs were conducted to assess differences between groups on the delta of performance of either short-term (hypothesis 2.a), long-term (hypothesis 2.a) or retention (hypothesis 3), each time controlling for video game experience.

Whenever significant effect of the group was observed on previous ANCOVAs, we performed post-hoc comparisons to assess the differences between each groups (hypothesis 4). P-values have been adjusted using the Holm correction^75^. To measure the effect size, we computed the $\eta _p^2$. We specified the assessed Beta value (referred to as b in the text) for the co-variate results to indicate the direction (positive or negative) of the effect.

## Supplementary Information

Supplementary Information.

## Data Availability

The raw data supporting the conclusions of this article will be made available by the authors, without undue reservation.
