# Supplementary material for: Performance after training in a complex cognitive task is enhanced by high-definition transcranial random noise stimulation
Source: Sci Rep. 2022 Mar 17;12:4618. doi: 10.1038/s41598-022-08545-x (PMC8931133; doi:10.1038/s41598-022-08545-x)
Supplement: Supplementary file 1 — Supplementary Information. [file 41598_2022_8545_MOESM1_ESM.pdf]

Supplementary Material

Tables

Table S 1. Score distribution of Space Fortress sub-tasks.

Sub-task	Keyboard*	Rules	Points
1. Controlling the ship	z, q, d	- Avoid being hit	-50
		- Avoid being destroyed (after 3 hits)	-100
		- Avoid crossing game borders	-35
		- Avoid colliding with the Space Fortress	-35
		- Manage the missile stock	None
2. Destroying the fortress	spacebar	- Hit the fortress 10 times with at least 250 ms between each shot	None
		- Destroy the fortress with a double shot (after 10 hits)	250
3. Destroying the mines	j	- Memorize three letters at the beginning	None
		- Identify Type-1 or Type-2 mines depending on the letters	None
		- Destroy a mine	50 or 60
		- Fail to destroy a mine before it disappears	-50
4. Capturing bonuses	k, l	- Check the appearance of random pairs of symbols, one symbol at a time (# $ & or @)	None
		- If the pair is # then $, capture the bonus	50 or 100
		- Fail to capture a bonus	-50

*The experiment was conducted on an AZERTY computer keyboard.

Table S 2. Summary of tRNS studies that investigated its effect on cognition (part 1/2)

Authors	Aim	N	Groups	Task	Cognitive function	Training	Sponge Size	Localization	tES frequencies	tES intensity	Stimulation time	Result
Mulquiney et al., 2011[1]	Investigate if WM can be improved after tRNS/tDCS (comparison)	10	Three sessions (within): tDCS vs tRNS vs Sham	Sternberg WM task	Working memory	No	7x5 cm	anode F3 and supraorbital	101-640 Hz	-05 to 0,5 mA	10 min (ramp 20 sec)	No effect of tRNS (but performance improvement for tDCS)
Snowball et al., 2013[2]	Investigate if tRNS can improve learning and performance on complex arithmetic task	25	Two groups: tRNS (13) vs Sham (12)	arithmetic task	Numerical manipulation	Yes - 5 x 20 min	5x5 cm	F3 and F4	100-600 Hz	-0,5 to 0,5 mA	20 min (ramp 15 sec)	Improved learning rate and performance for the tRNS group, even in the long-lasting session (6 months)
Holmes et al., 2016[3]	Investigate if cognitive training with brain stimulation (tRNS) improve working memory (transfer)	30	Two groups: tRNS (15) vs Sham (15)	n-back	Working memory	Yes - 10 x 45 min (Cogmed)	5x5 cm	anode F4, cathode P4 (bilateraly)	101-640 Hz	-0,5 to 0,5 mA	20 min (ramp 15 sec)	No effect of tRNS
Pasqualotto et al., 2016[4]	Compare tRNS montages on an arithmetical task performance	54	Three groups: Frontal (18) vs Parietal (18) vs Sham (18) stimulation	arithmetics (addition, multiplications, etc)	Numerical manipulation	No	5x5 cm	frontal or parietal	100-600 Hz	1 mA	20 min (ramp 10 sec)	Improved performance for the tRNS group in the long-term session (7 days later)
Popescu et al., 2016[5]	Investigate the learning efficiency in arithmetics using tRNS + cognitive training	32	Two groups: tRNS (16) vs Sham (16)	calculation task	Numerical manipulation	Yes - 5 x 20 min	4x4 cm	F3/F4 then P3/P4	100-640 Hz	-0,5 to 0,5 mA	20 min (ramp 15 sec)	Improved performance and transfer for the tRNS group
Bieck et al., 2018[6]	Compare tRNS montages on an arithmetical task performance	48	Three sessions (within): Frontal vs Parietal vs Sham stimulation	two digit addition	Numerical manipulation	No	5x5 cm	P3/P4 (IPS) and F3/F4 (DLPFC)	100-640 Hz	-0,5 to 0,5 mA	20 min (ramp 15 sec)	No effect of tRNS
Brauer et al., 2018[7]	Compare different tES montages on go/nogo task performance	23	Three sessions (within): tACS vs TRNS vs Sham stimulation	Go/No Go task	Inhibition	No	5x5 cm	Fp1 (IFG) and between T4-Fz and F8-Cz	0,1-640Hz	1 mA	20 min (ramp 10 sec)	No effect of tRNS/tACS
Brem et al., 2018[8]	Investigate if cogntive training coupled with brain stimulation montages (tDCS / tRNS / mftACS / mftDCS / SHAM) improve fluid intelligence (transfer)	82	Five groups: tDCS (17) vs tRNS (16) vs mtDCS (15) vs tACS (17) vs Sham (17)	Raven's matrices	Fluid intelligence	Yes - 9 x 30 min (Video game)	3,14x3,14 cm	anode F3	Not specified	-0,5 to 0,5 mA	30 min (ramp 30 sec)	Transfer effect for the tDCS / mftDCS / tRNS groups (not for mftACS / SHAM)
Brevet et al., 2019[9]	Investigate the effect of tRNS on the Go/NoGo task performance (short and long-term)	33	Three groups: 3 sham sessions (11) vs 1 tRNS + 2 sham sessions (10) vs 3 tRNS sessions (12)	Go/No Go task	Inhibition	Yes - 3 x 30 min	7x5 cm	anode F3, cathode F4	100-500 Hz	2 mA	20 min (ramp 30 sec)	No short term effect, but a long-term effect (8-day follow up) of the tRNS group with improved performance

Summary of tRNS studies that investigated its effect on cognition (part 2/2)

Authors	Aim	N	Groups	Task	Cognitive function	Training	Sponge Size	Localization	tES frequencies	tES intensity	Stimulation time	Result
Dondé et al., 2019[10]	investigate the effect of a single tRNS session over the DLPFC on stroop performance	19	Two groups: tRNS (8) vs Sham (11)	Stroop	Inhibition	No	7x5 cm	anode F4, cathode F3	100-500 Hz	2 mA	20 min (ramp 30 sec)	No effect of tRNS
Frank et al., 2018[11]	investigate the effects of tRNS over the learning of a complex task	40	Two groups: tRNS (20) vs Sham (20)	start-up procedure of simulated waste water treatment	executive functions	No	5x5 cm	anode F4, cathode F3	100-500 Hz	1 mA	12 min (ramp 30 sec)	Improved performance for the tRNS on the short- and long-term (but mediated by general mental abilities, e.g. beneficial for those with lower general abilities)
Harty et al., 2019[12]	Investigate the effects of tRNS on sustained attention and theta/beta ratio marker	72	Three sessions (within): 2 mA vs 1 mA vs Sham	continuous monitoring task	Attention	No	5x5 cm	anode F4, cathode P4	100-500 Hz	-0,5 to 0,5 mA and -1 to 1 mA	20 min (ramp 30 sec)	Improved performance for the 1 mA vs sham & 2 mA; explained by a reduction in the theta/beta ratio
Krause et al., 2019[13]	investigate tRNS on high functioning individual vs healthy control	7	Two groups: world champion in mental calculation (1) vs not calculation prodiges (6)	mathematical multiplication	Numerical manipulation	No	5x5 cm	F3 and F4	0,1-500 Hz	1 mA	20 min (ramp 15 sec)	No effect of tRNS
Almquist et al., 2019[14]	Investigate if cognitive training with brain stimulation (tRNS) improve fluid intelligence (transfer)	91	Three groups: tRNS+cog training (32) vs Sham+control task (30) vs no training (29)	Advanced Progressive Matrices	Fluid intelligence	Yes - 10 x 30 min (video game)	3,14x3,14 cm	F3 and F4	100-500 Hz	1 mA	20 min (ramp 30 sec)	Improved performance for the tRNS group
Murphy et al., 2020[15]	Compare the effects of tDCS, tRNS on WM performance	49	Three groups: tDCS (16) vs tRNS (16) vs Sham (17)	Sternberg task	Working memory	No	sponge 7x5 cm	anode F3 and supraorbital	100-640 Hz	0,5 to 1,5 mA	22 min (ramp 60 sec)	Improved performance for the tRNS group compared to the Sham/tDCS

Figures

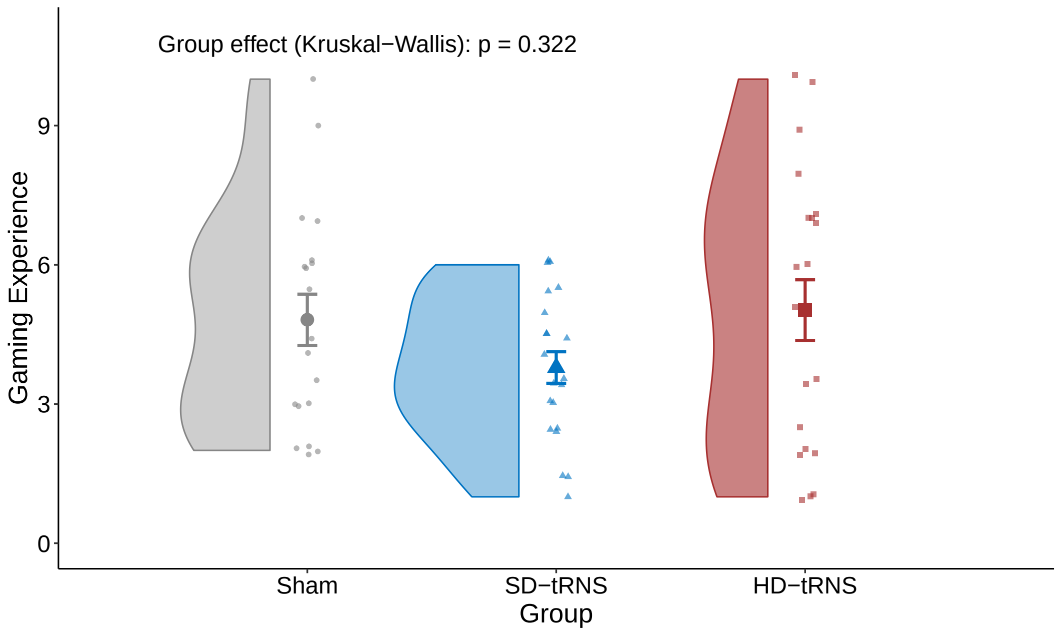

Figure S 1. Gaming experience by Group. This index is calculated from 3 questions that assess the frequency and intensity of past and present game use. Score ranges from 0 (no gaming at all) up to 10 (heavy gaming).

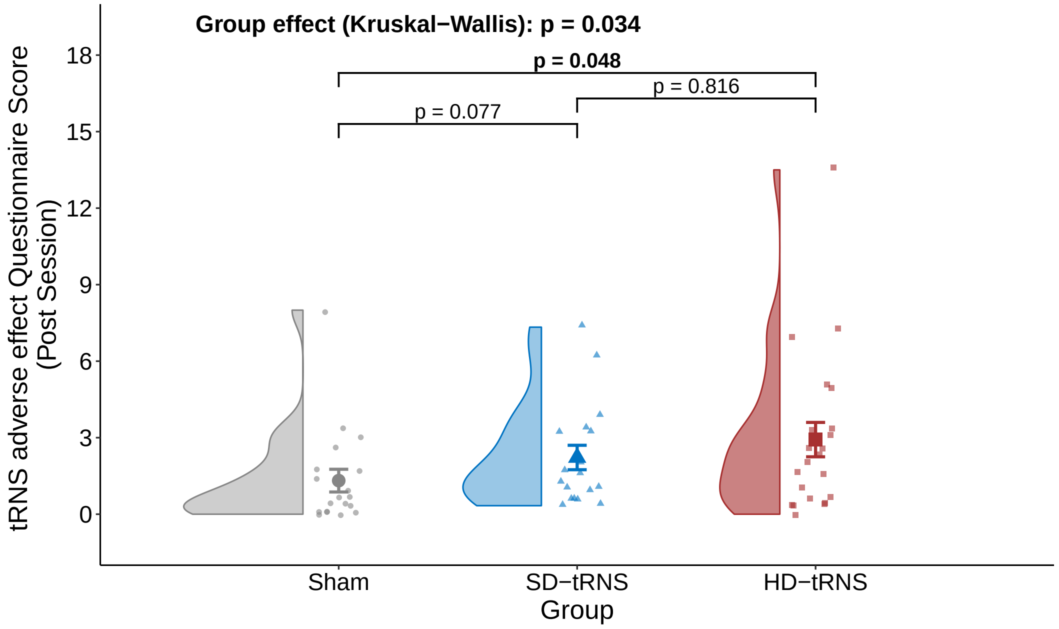

Figure S 2. Score on the post-session tRNS Adverse Effect Questionnaire per group (mean of the three training sessions with stimulation). **Bold:** $p < .05$

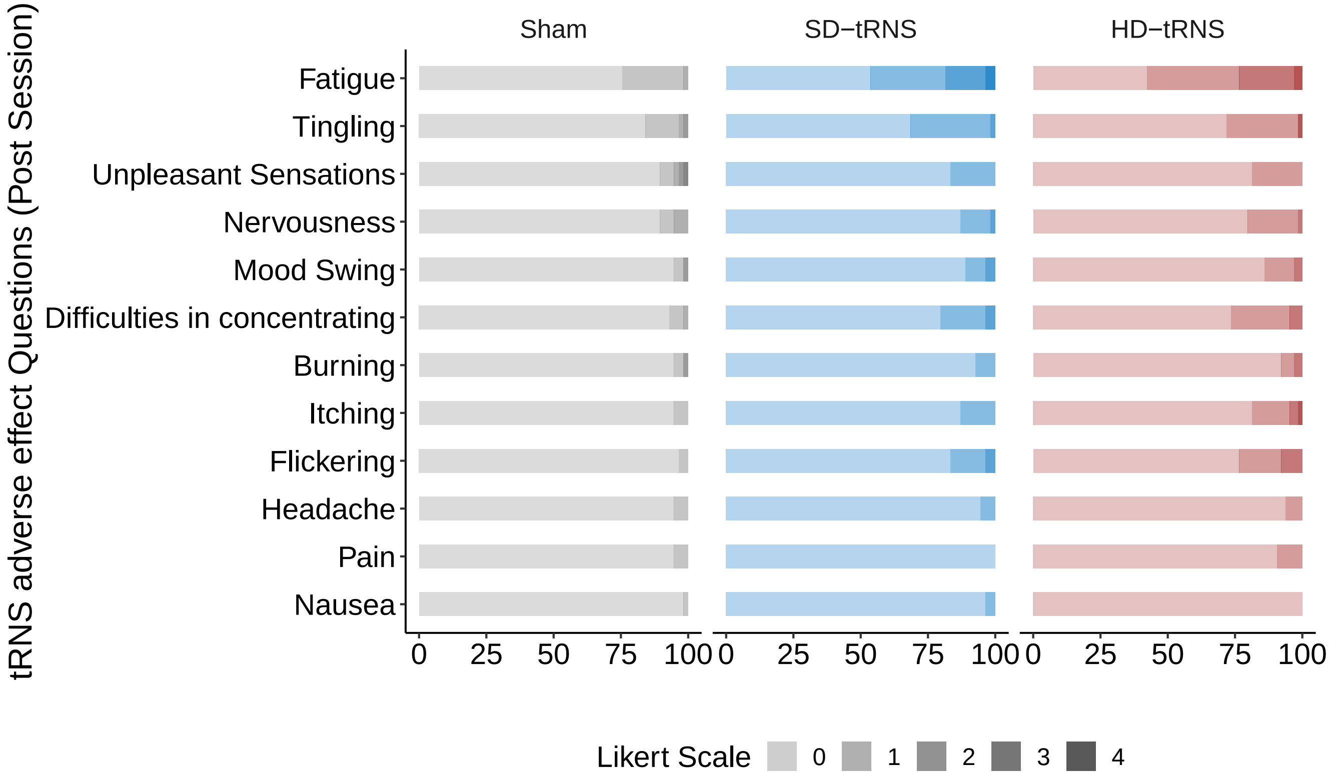

Figure S 3. Percentage barplot of each item of tRNS Adverse Effect Questionnaire (post session)

A priori power analysis

The *a priori* power analysis was computed based on the results from a pilot study with two groups of 5 participants which was published as a poster in the CORTICO conference (Lille, France, 2019, https://www.researchgate.net/publication/346084303_Random_noise_brain_stimulation_for_video_game_learning). With this sample, we showed a large effect size in the long-term retention (Cohen's d = .8) for the tRNS group *vs* the sham group (only SD montages). The *a priori* power analysis (ANOVA) was performed with the r software and with the package "pwr" (*pwr.anova.test* function) using the following parameters: (1) Effect size (f) = .4 (because the sample in the pilot study was limited, there was a high risk of effect size inflation. Therefore, we used an effect size only half of what we observed); (2) Number of groups (k) = 3 (because we added the HD-tRNS group); (3) p (*sig.level*) = .05; (4) *power* = .8 (as recommended in the literature, see Button et al.[16]). The function gave in return the number of participants to be recruited (here, n = 21.1). Based on this analysis, we decided to recruit 22 participants per group.

References

1. Mulquiney, P. G., Hoy, K. E., Daskalakis, Z. J. & Fitzgerald, P. B. Improving working memory: exploring the effect of transcranial random noise stimulation and transcranial direct current stimulation on the dorsolateral prefrontal cortex. *Clin. Neurophysiol.* **122**, 2384–2389 (2011).
2. Snowball, A. *et al.* Long-term enhancement of brain function and cognition using cognitive training and brain stimulation. *Curr. Biol.* **23**, 987–992 (2013).
3. Holmes, J., Byrne, E. M., Gathercole, S. E. & Ewbank, M. P. Transcranial random noise stimulation does not enhance the effects of working memory training. *J. Cogn. Neurosci.* **28**, 1471–1483 (2016).

4. Pasqualotto, A. Transcranial random noise stimulation benefits arithmetic skills. *Neurobiol. learning memory* **133**, 7–12 (2016).

5. Popescu, T. *et al.* Transcranial random noise stimulation mitigates increased difficulty in an arithmetic learning task. *Neuropsychologia* **81**, 255–264 (2016).

6. Bieck, S. M., Artemenko, C., Moeller, K. & Klein, E. Low to no effect: application of trns during two-digit addition. *Front. neuroscience* **12**, 176 (2018).

7. Brauer, H., Kadish, N. E., Pedersen, A., Siniatchkin, M. & Moliadze, V. No modulatory effects when stimulating the right inferior frontal gyrus with continuous 6 hz tacs and trns on response inhibition: a behavioral study. *Neural plasticity* **2018** (2018).

8. Brem, A.-K. *et al.* Modulating fluid intelligence performance through combined cognitive training and brain stimulation. *Neuropsychologia* **118**, 107–114 (2018).

9. Brevet-Aeby, C., Mondino, M., Poulet, E. & Brunelin, J. Three repeated sessions of transcranial random noise stimulation (trns) leads to long-term effects on reaction time in the go/no go task. *Neurophysiol. Clinique* **49**, 27–32 (2019).

10. Dondé, C., Brevet-Aeby, C., Poulet, E., Mondino, M. & Brunelin, J. Potential impact of bifrontal transcranial random noise stimulation (trns) on the semantic stroop effect and its resting-state eeg correlates. *Neurophysiol. Clinique* **49**, 243–248 (2019).

11. Frank, B., Harty, S., Kluge, A. & Cohen Kadosh, R. Learning while multitasking: short and long-term benefits of brain stimulation. *Ergonomics* **61**, 1454–1463 (2018).

12. Harty, S. & Cohen Kadosh, R. Suboptimal engagement of high-level cortical regions predicts random-noise-related gains in sustained attention. *Psychol. science* **30**, 1318–1332 (2019).

13. Krause, B., Dresler, M., Looi, C. Y., Sarkar, A. & Kadosh, R. C. Neuroenhancement of high-level cognition: evidence for homeostatic constraints of non-invasive brain stimulation. *J. Cogn. Enhancement* **3**, 388–395 (2019).

14. Almquist, J. N.-F. *et al.* Fast: A novel, executive function-based approach to cognitive enhancement. *Front. human neuroscience* **13**, 235 (2019).

15. Murphy, O. *et al.* Transcranial random noise stimulation is more effective than transcranial direct current stimulation for enhancing working memory in healthy individuals: Behavioural and electrophysiological evidence. *Brain Stimul.* **13**, 1370–1380 (2020).

16. Button, K. S. *et al.* Power failure: why small sample size undermines the reliability of neuroscience. *Nat. reviews neuroscience* **14**, 365–376 (2013).
